# Editorial: Production of novel medical radionuclides and innovative radiopharmaceuticals

**DOI:** 10.3389/fchem.2024.1386045

**Published:** 2024-02-27

**Authors:** Susanta Lahiri

**Affiliations:** ^1^ Department of Chemistry, Diamond Harbour Women’s University, Sarisha, India; ^2^ Department of Physics, Sidho Kanho Birsha University, Purulia, India

**Keywords:** cyclotron production, theragnostic radioisotopes, Sc radioisotopes, Cu radioisotopes, Lu radioisotopes, Tm radioisotopes

It has been more than 100 years and radioactivity and radioisotopes are still serving mankind. The aim of nuclear science, nuclear physics, nuclear chemistry, or nuclear medicine is to discover new radioactive isotopes for the betterment of mankind, to design benign separation methods of no-carrier-added radioisotopes from the target matrix, and to synthesize new radiopharmaceuticals for more precise delivery of the radioisotope to the target organs.

Nuclear medicine passed through several phases. Two important focuses in the present phase are 1) to discover theragnostic pair of radionuclides, which will simultaneously perform therapy and diagnosis and reduce radiation burden to patient and operators and 2) to investigate non-standard (different from most common ones) radioisotopes for diagnosis and therapy. These non-standard radioisotopes provide opportunities for personalized medicine. New insights in the radiolabeling methods made the present phase of nuclear medicine more human-centric, and radiopharmaceuticals are designed with human-compatible biomolecules like DOTA-TATE (tyrosine analogue), DOTATOC, somatostatin, human monoclonal antibody, etc.

In this Research Topic of Frontiers in Chemistry, “*Production of Novel Medical Radionuclides and Innovative Radiopharmaceuticals*” stalwarts in the field described the current state of knowledge on different Medical Radionuclides. There are four articles on the clinical importance of the radionuclides of four elements, Sc, Cu, Tm and Lu, distributed over various periods and groups of the Periodic Table.

The Sc radionuclides ^43^Sc, ^44g^Sc, and ^47^Sc are being considered as alternatives to Ga radionuclides. ^43^Sc/^44g^Sc and ^47^Sc combination may be good theragnostic pairs. However, to date, the production of Sc radionuclides, in the playable amount required for *in vivo* administration, has several constraints including the use of costly enriched targets. The article by Becker et al. describes various aspects of cyclotron production of Sc radionuclides and reviews the current state of knowledge, and using Sc radionuclides as an alternative to Ga may be helpful in the future.

The copper radionuclides are now in the centre stage of discussion for *in vivo* theragnostic applications. ^60,61,62,64^Cu radionuclides are positron emitters and therefore suitable for imaging. Out of these, ^60^Cu and ^62^Cu have relatively shorter half-lives and *in vivo* application is possible only at a very near vicinity of the cyclotron. The other two radionuclides, i.e., ^61^Cu and ^64^Cu have considerable half-lives for imaging purposes. On the other hand, ^67^Cu is a $\beta^{-}$ emitter and suitable for therapeutic application. Therefore, ^67^Cu and any one of the four imaging radionuclides of copper together will be an excellent theragnostic pair of radionuclides. ^64^Cu is another interesting radionuclide; it emits Auger electron, $\beta^{-}$ , and $\beta^{+}$ . Therefore, this single radionuclide can be used for theragnostic purposes. However, the literature review reveals lots of discrepancies in nuclear data in terms of thick target yield, excitation function, and decay data. These discrepancies would lead to erroneous calculations and would ultimately affect the calculation of radiation dose to target tissues. The article by Hussain et al. authoritatively discusses the discrepancies in the nuclear data and is expected to serve the nuclear scientists including physicians for a long time to carry out the intended clinical task.

In the last decade, the most successful “lab to bed” radioisotope is ^177^Lu, which has been regularly supplied to hospitals for the treatment of various types of ailments related to cancer including neuroendocrine tumours and prostate cancer. Several vectors like DOTA-TATE, PSMA, etc., are tagged to ^177^Lu for making the radioisotope target-specific. George and Samuel presented a comprehensive review of the current status of the radiopharmaceutical chemistry of ^177^Lu as well as personalized dosimetry upon delivering ^177^Lu to the patients.

The article by Renaldin et al. highlights another lanthanide radionuclide ^167^Tm. It is true that the other two lanthanide radionuclides, ^177^Lu and the terbium quadruplets, ^149,152,155,161^Tb have gotten more attention in recent times. Nevertheless, the nuclear properties of ^167^Lu would allow it to follow longer biological processes. The authors compared the theoretical simulation codes and experimental production cross-sections of ^167^Tm from various target materials like enriched ^167^Er, ^168^Er, ^nat^Tm, ^nat^Yb, etc. They also discussed the radiochemical separation technique of ^167^Tm from the target matrix.

In a nutshell, I believe that this Research Topic of Frontiers in Chemistry would be a reference for both experts and beginners.

Thanks to my co-editors Dr. Zeynep Talip and Dr. Kohshin Washiyama. Thanks also to the Frontiers team for all the help to bring out this nice issue.

